# Supplementary material for: Analysis of PPARγ Signaling Activity in Psoriasis
Source: Int J Mol Sci. 2021 Aug 10;22(16):8603. doi: 10.3390/ijms22168603 (PMC8395241; doi:10.3390/ijms22168603)
Supplement: Supplementary file 1 [file ijms-22-08603-s001.zip › Supplemental materials_Analysis of PPARg signaling activity in psoriasis/Pathway models/Models images and html files/Interleukin-17 and interleukin-22 signaling in psoriasis/10347.html]

CCL7


# Protein CCL7

|  |  |
| --- | --- |
| URN | urn:agi-llid:6354 |
| Total Entities | 0 |
| Connectivity | 1088 |
| Name | CCL7 |
| Description | C-C motif chemokine ligand 7 |
| Notes | This gene encodes monocyte chemotactic protein 3, a secreted chemokine which attracts macrophages during inflammation and metastasis. It is a member of the C-C subfamily of chemokines which are characterized by having two adjacent cysteine residues. The protein is an in vivo substrate of matrix metalloproteinase 2, an enzyme which degrades components of the extracellular matrix. This gene is part of a cluster of C-C chemokine family members on chromosome 17q. [provided by RefSeq, Jul 2008] |
| Primary Cell Localization | Extracellular |
| Class | Ligand |

---

|  |  |
| --- | --- |
| Pathway | CC Chemokine Receptor Signaling |
|  | Mast-Cell Activation via IgE Signaling |
|  | Nociception Expression Targets Signaling |
|  | CCR1 Expression Targets |
|  | Proteins Involved in Atherosclerosis |
|  | Proteins Involved in Myocarditis |
|  | IL17 Signaling in Psoriasis |
|  | Mast-Cell Activation in Asthma |
|  | Cytokines Trigger Otitis Media |
|  | CCL7 Expression Targets |
|  | F2R/F2RL1 -> Expression Targets in Lymphoid System and Blood |
|  | 2\_Interleukin-17 and interleukin-22 signaling in psoriasis |
|  | Interleukin-17 and interleukin-22 signaling in psoriasis |

---

|  |  |
| --- | --- |
| Group | Genes with Mutations Associated with Crohn's Disease |
|  | Chemokines, Growth Factors and Receptors Involved in Cancer Cells Chemotaxis |

---

|  |  |
| --- | --- |
| MedScan ID | 6354 |

---

|  |  |
| --- | --- |
| LocusLink ID | 6354 |

---

|  |  |
| --- | --- |
| Alias | chemokine (C-C motif) ligand VII |
|  | SCYA6 |
|  | MGC138463 |
|  | SCYA7 |
|  | chemotactic protein-3 |
|  | MGC138465 |
|  | SY07\_HUMAN |
|  | CCL7 |
|  | Monocyte chemotactic protein 3 |
|  | MCP-3 |
|  | C-C motif chemokine 7 |
|  | SY07 |
|  | fic |
|  | CCL7 chemokine |
|  | chemokine CCL7 |
|  | MCP III |
|  | Monocyte chemoattractant protein 3 |
|  | chemotactic protein III |
|  | NC28 |
|  | CC chemokine ligand 7 |
|  | small inducible cytokine A7 |
|  | Monocyte chemotactic protein III |
|  | MCP IIIs |
|  | small inducible cytokine A7 (monocyte chemotactic protein 3) |
|  | Monocyte chemoattractant protein III |
|  | OTTMUSP00000000787 |
|  | C-C motif chemokine ligand 7 |
|  | RP23-350G1.4 |
|  | MARC |
|  | chemokine (C-C motif) ligand 7 |
|  | CCL-7 |
|  | MCP3 |
|  | small-inducible cytokine A7 |

---

|  |  |
| --- | --- |
| GO ID | 0048020 |
|  | 0031726 |
|  | 0031727 |
|  | 0008009 |
|  | 0008201 |
|  | 0007186 |
|  | 0007267 |
|  | 0006874 |
|  | 0071361 |
|  | 0071346 |
|  | 0071347 |
|  | 0071356 |
|  | 0070098 |
|  | 0006935 |
|  | 0007010 |
|  | 0048245 |
|  | 0006954 |
|  | 0048247 |
|  | 0002548 |
|  | 0030593 |
|  | 0070374 |
|  | 0043547 |
|  | 0030335 |
|  | 2000503 |
|  | 0008360 |
|  | 0010332 |
|  | 0007165 |
|  | 0005623 |
|  | 0005615 |
|  | 0050729 |
|  | 0005576 |
|  | 0006955 |

---

|  |  |
| --- | --- |
| KEGG ID | hsa:6354 |

---

|  |  |
| --- | --- |
| Organism | Homo sapiens {Organism urn:agi-taxid:9606} |
|  | Homo sapiens |

---

|  |  |
| --- | --- |
| OMIM ID | 158106 |

---

|  |  |
| --- | --- |
| Hugo ID | 10634 |
|  | HGNC:10634 |

---

|  |  |
| --- | --- |
| Human chromosome position | 17q12 |
|  | 17q11.2-q12 |

---

|  |  |
| --- | --- |
| Swiss-Prot Accession | P80098 |
|  | P80098.3 |
|  | Q569J6 |

---

|  |  |
| --- | --- |
| GenBank ID | NC\_000017 |
|  | NM\_006273 |
|  | NP\_006264 |
|  | AC005549 |
|  | CH471147 |
|  | EAW80210 |
|  | X72309 |
|  | CAB59723 |
|  | X95070 |
|  | AF043338 |
|  | AAC03538 |
|  | BC070240 |
|  | BC092436 |
|  | AAH92436 |
|  | BC112258 |
|  | AAI12259 |
|  | BC112260 |
|  | AAI12261 |
|  | CD523198 |
|  | X71087 |
|  | CAA50405 |
|  | CAA50406 |
|  | CAA50407 |
|  | P80098 |
|  | NC\_018928 |
|  | AMYH02033336 |
|  | HQ258695 |
|  | ADR83446 |
|  | AC\_000149 |
|  | ABBA01011212 |

---

|  |  |
| --- | --- |
| Swiss-Prot ID | CCL7\_HUMAN |

---

|  |  |
| --- | --- |
| Cell Localization | Secreted |

---

|  |  |
| --- | --- |
| Ensembl ID | ENSG00000108688 |
|  | ENSP00000367832.2 |
|  | ENST00000378569.2 |
|  | ENSP00000367832 |
|  | ENST00000378569 |

---

|  |  |
| --- | --- |
| Unigene ID | Hs.251526 |

---

|  |  |
| --- | --- |
| Shape | Rhomb |

---
